# The Dynamics and Regulatory Mechanism of Pronuclear H3k9me2 Asymmetry in Mouse Zygotes

**DOI:** 10.1038/srep17924

**Published:** 2015-12-07

**Authors:** Xue-Shan Ma, Shi-Bin Chao, Xian-Ju Huang, Fei Lin, Ling Qin, Xu-Guang Wang, Tie-Gang Meng, Cheng-Cheng Zhu, Heide Schatten, Hong-Lin Liu, Qing-Yuan Sun

**Affiliations:** 1 State Key Laboratory of Reproductive Biology, Institute of Zoology, Chinese Academy of Sciences, Beijing 100101, China; 2 College of Animal Science and Technology, Nanjing Agricultural University, Nanjing 210095, China; 3 The ART Center of Jiujiang Maternal and Child Health Care Hospital, Jiujiang 332000, China; 4 College of Animal Science, Xinjiang Agricultural University, Xinjiang 830025, China; 5 University of Chinese Academy of Sciences, Beijing 100101, China; 6 Department of Veterinary Pathobiology, University of Missouri, Columbia, MO 65211, USA

## Abstract

H3K9 methylation is an important histone modification that is correlated with gene transcription repression. The asymmetric H3K9 dimethylation (H3K9me2) pattern between paternal and maternal genomes is generated soon after fertilization. In the present study, we carefully determined the dynamics of H3K9me2 changes in mouse zygotes, and investigated the regulatory mechanisms. The results indicated that histone methyltransferase G9a, but not GLP, was involved in the regulation of asymmetric H3K9me2, and G9a was the methyltransferase that induced the appearance of H3K9me2 in the male pronucleus of the zygote treated with cycloheximide. We found that there were two distinct mechanisms that regulate H3K9me2 in the male pronucleus. Before 8 h of *in vitro* fertilization (IVF), a mechanism exists that inhibits the association of G9a with the H3K9 sites. After 10 h of IVF the inhibition of G9a activity depends on yet unknown novel protein(s) synthesis. The two mechanisms of transfer take place between 8–10 h of IVF, and the novel protein failed to inhibit G9a activity in time, resulting in the appearance of a low level *de novo* H3K9me2 in the male pronucleus.

Epigenetic alterations are heritable changes that are not encoded by DNA sequences in a cell, and such changes can be delivered stably throughout development and cell proliferation. Epigenetic mechanisms are essential for normal development and maintenance of tissue-specific gene expression patterns in mammals^1^. Thus, a comprehensive understanding of epigenetic mechanisms, their interactions and alterations, has become a high research priority^2^. The methylation of histones on different lysine sites results in different biological effects^3^. Generally, H3K9, H3K27 and H4K20 methylations are correlated with gene transcription repression, whereas H3K4 and H3K36 methylations are correlated with gene transcription activation^4^,^5^,^6^,^7^,^8^,^9^. The H3K9 site can have single, double and triple methylation, and different degrees of methylation results in different distributions and functions. H3K9 methylation catalyzed by histone-lysine-methyltransferases involves transcriptional silencing and heterochromatin formation. For all model species, the heterochromatin protein HP1 or HP1 homologue show high affinity with the H3K9 methylation^10^,^11^.

In mammalian cells, H3K9 methyltransferase enzymes include SUV39H1^12^, SUV39H2^13^,^14^, Eu-HMTase/GLP^15^, G9a^16^, ESET/SETDB1^17^ and RIZ1^18^. SUV39H1, SUV39H2 and ESET are thought to catalyze H3K9 trimethylation (H3K9me3), while G9a and GLP catalyze H3K9 dimethylation (H3K9me2). G9a plays a catalytic role in H3K9me2 activity at euchromatin regions and causes gene expression inhibition^16^. Another euchromatin area displays histone methyltransferase GLP that plays a role in silencing of E2F- and Myc-responsive genes in quiescent cells^15^. G9a and GLP form heteromeric complexes and are linked by the zinc finger protein Wiz^19^,^20^,^21^. G9a and GLP play dominant roles in euchromatic histone H3K9 methylation and are essential for early embryogenesis; G9a- and GLP-deficient embryos display severe growth retardation and early lethality^19^,^22^.

There are several kinds of epigenetic alterations in mouse zygotes, including asymmetry of DNA methylation and a series of histone modifications between male and female pronuclei^23^. Therefore, the zygote is an excellent stage to study the epigenetic mechanisms. In this paper, we investigated the alterations and mechanisms of H3K9me2 in mouse zygotes. Previous studies have shown that the asymmetric H3K9me2 pattern between parental genomes is generated soon after fertilization. H3K9me2 showed a very weak or absent methylation signal in the male pronucleus, whereas a distinct methylation signal was detected in the female pronucleus^24^. Our study carefully examined H3K9me2 levels in different pronuclear stages of zygotes; we found that a low level *de novo* H3K9me2 occurred in the male pronucleus at 10 h of *in vitro* fertilization (IVF), while *de novo* H3K9me2 of zygotes treated with cycloheximide occurred at the same time, suggesting that there might be two distinct mechanisms that regulate the male pronucleus H3K9me2 in mouse zygotes.

## Results

### Alteration of H3K9me2 in mouse zygotes

**Figure 1:**
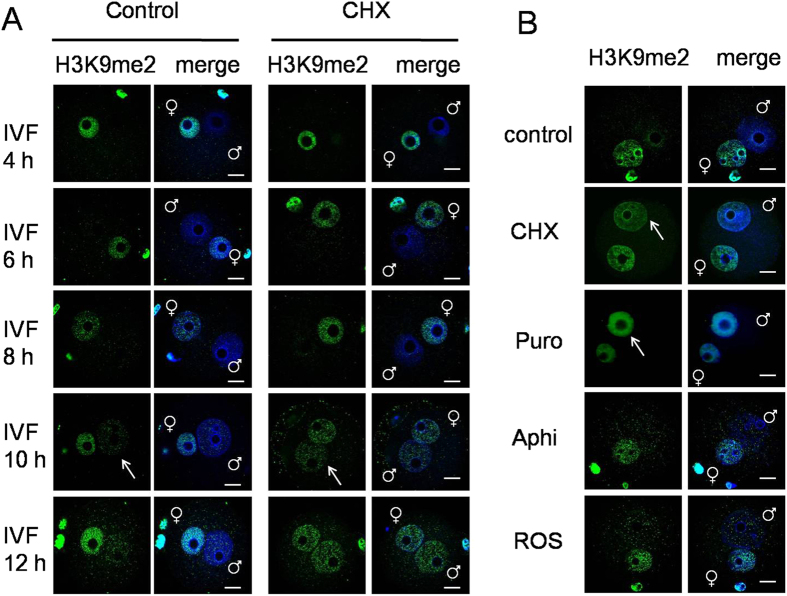
Alteration of H3K9me2 in mouse zygotes after different treatments. (**A**) The H3K9me2 state of zygotes at 4, 6, 8, 10 and 12 h of IVF detected by immunostaining. Control indicates zygotes cultured in normal KSOM medium, and CHX indicates zygotes cultured in KSOM medium containing cycloheximide. (**B**) The H3K9me2 state of zygotes at 12 h of IVF treated with different agents. CHX, Puro, ROS and Aphi indicate zygotes cultured in KSOM medium containing cycloheximide, puromycin, roscovitin and aphidicolin, respectively. Confocal micrographs show the immunostained H3K9me2 (green) and DNA (DAPI, blue) in mouse zygotes. White arrows indicate the increased H3K9me2 of the male pronucleus. Scale bar = 20 μm.

Fertilized eggs were collected at 4 h, 6 h, 8 h, 10 h and 12 h of IVF, respectively. After immunostaining with H3K9me2 antibody, the female pronucleus displayed high H3K9me2 levels in all stages (Fig. 1A). In contrast, the male pronucleus showed no H3K9me2 signal or only low H3K9me2 levels. These results are consistent with previous reports^24^,^25^,^26^,^27^. However, we noticed that there was a low but distinct H3K9me2 signal in the male pronucleus after 10 h of IVF; in contrast, almost no H3K9me2 signal was observed before 8 h of IVF. When treated with cycloheximide, the male pronucleus showed increased H3K9me2 to a level that was similar to that of the female pronucleus after 10 h of IVF (indicated with white arrow, Fig. 1A).

We further investigated the effect of inhibition of protein synthesis, DNA replication and protein kinase on H3K9me2 in mouse zygotes, as shown in (Fig. 1B). Cycloheximide and puromycin are two protein synthesis inhibitors with different mechanisms^28^. Roscovitine is a purine analog that is a potent and selective inhibitor of cyclin-dependent kinases (CDK)^29^,^30^. Aphidicolin is an inhibitor of DNA replication hat specifically inhibits DNA polymerase α^31^. Fertilized eggs were transferred at 2h of IVF into KSOM medium containing different reagents, and collected at 12 h of IVF. Immunostaining revealed that H3K9me2 levels in zygotes treated with cycloheximide and puromycin displayed a clear increase, especially in the male pronucleus (indicated with white arrow, Fig. 1B), suggesting that the asymmetric H3K9 methylation pattern between paternal and maternal genomes disappeared after inhibiting protein synthesis. Meanwhile, the H3K9me2 level of zygotes treated with aphidicolin and roscovitin showed no significant difference compared with the control group.

### BIX 01294 inhibited the increase of H3K9me2 in mouse zygotes

**Figure 2:**
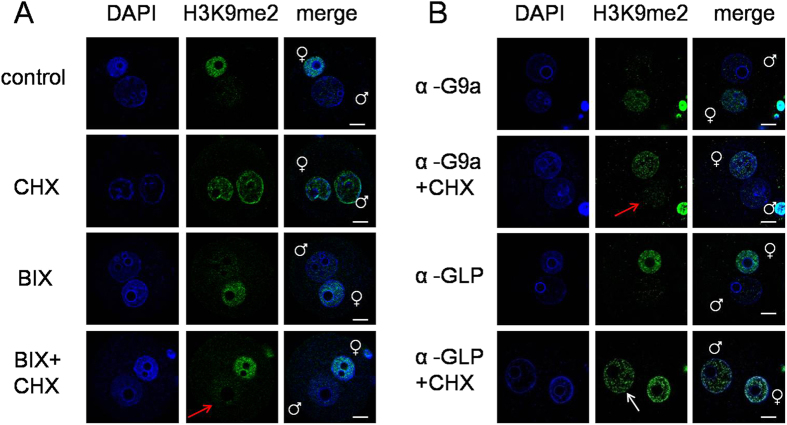
The influence of BIX 01294 and G9a or GLP antibody microinjection on H3K9me2. (**A**) The H3K9me2 state of zygotes at 12 h of IVF treated with BIX 01294. CHX and BIX indicate zygotes cultured in KSOM medium containing cycloheximide and BIX01294, respectively. BIX+CHX indicates zygotes cultured in KSOM medium containing cycloheximide and BIX01294 together. (**B**) The H3K9me2 state of zygotes at 12 h of IVF after G9a or GLP antibody microinjection. α -G9a and α –GLP indicates zygotes cultured in normal KSOM medium after antibody microinjection. And α -G9a+CHX and α –GLP+CHX indicates zygotes cultured in KSOM medium containing cycloheximide after antibody microinjection. White arrow indicates the increased H3K9me2 of the male pronucleus, and red arrows indicate the low H3K9me2 level of the male pronucleus. Scale bar = 20 μm.

The above results showed that the H3K9me2 level of the euchromatin area in the male pronucleus was dramatically increased after protein synthesis inhibition. G9a and GLP were the primary histone methyltransferases with catalytic H3K9me2 activity at euchromatin regions. We first investigated the effect of BIX 01294, a selective inhibitor of G9a and GLP, on H3K9me2 levels in mouse zygotes^32^,^33^. The H3K9me2 level of zygotes treated with BIX 01294 showed no significant difference with the control group (Fig. 2A). The H3K9me2 level of the male pronucleus in mouse zygotes treated with both cycloheximide and BIX 01294 did not increase (indicated with red arrow, Fig. 2A). This result indicates that BIX 01294 inhibits the catalytic H3K9me2 activity of histone methyltransferase in mouse zygotes treated with cycloheximide.

### G9a, but not GLP, was involved in the increase of H3K9me2 in mouse zygotes

**Figure 3:**
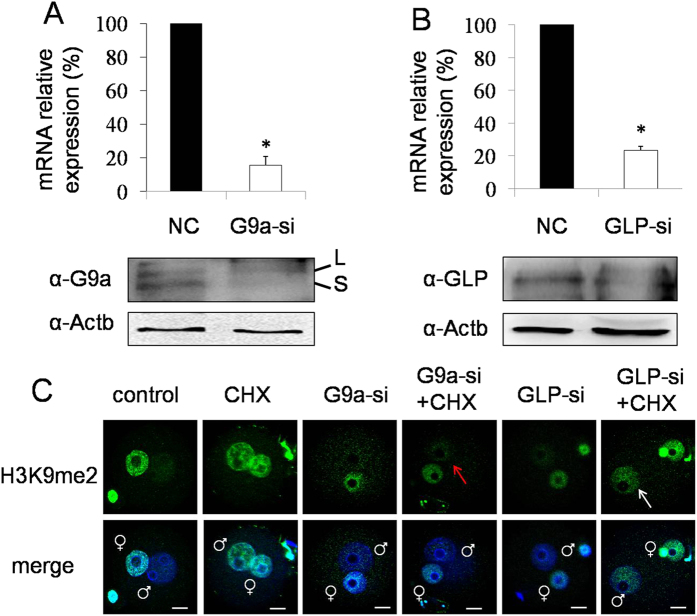
The influence of G9a or GLP siRNA microinjection on H3K9me2. (**A**) The mRNA and protein expression of G9a were detected after 24 h of G9a siRNA microinjection (G9a-si). L, the G9a long isoform. S, the G9a short isoform. *P < 0.01. (**B**) The mRNA and protein expression of GLP were detected after 24 h of GLP siRNA microinjection (GLP-si). Actb (beta-actin) was used as a loading control. *P < 0.01. (**C**) The H3K9me2 state of zygotes at 12 h of IVF after G9a or GLP siRNA microinjection. NC (negtive control) indicates zygotes microinjected with scrambled control siRNA. NC+CHX, G9a-si+CHX and GLP-si+CHX indicate zygotes cultured in KSOM medium containing cycloheximide after siRNA microinjection. White arrow indicates the increased H3K9me2 of the male pronucleus, and red arrow indicates the low H3K9me2 level of the male pronucleus. Scale bar = 20 μm.

Next, we investigated the function of G9a and GLP in mouse zygotes treated with cycloheximide, by microinjection of antibody, siRNA and mRNA. G9a or GLP antibody was microinjected into the cytoplasm of fertilized eggs at 2 h of IVF, then cultured for 10 h in KSOM medium containing cycloheximide. Immunostaining showed that asymmetric H3K9me2 disappeared in zygotes microinjected with G9a antibody and treated with cycloheximide (indicated with red arrow, Fig. 2B). But in the GLP antibody microinjection group, the asymmetric H3K9me2 still persisted after treatment with cycloheximide (indicated with white arrow, Fig. 2B). We further confirmed this phenotype by siRNA injection. The interference efficiency of G9a and GLP siRNA were detected by both quantitative real time-PCR and western blot analysis. G9a and GLP siRNA significantly reduced the expression of mRNA and protein, respectively (Fig. 3A,B). Importantly, similar results were obtained with G9a and GLP siRNA microinjection (indicated with red and white arrow, Fig. 3C).

**Figure 4:**
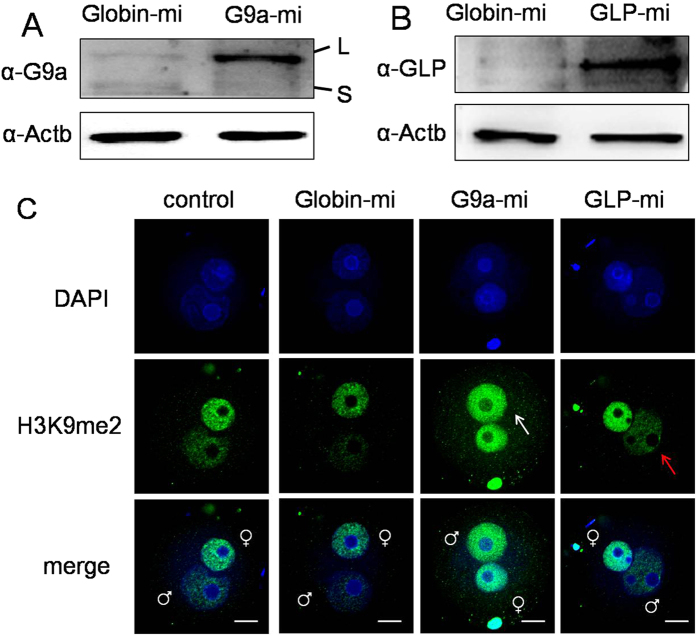
The influence of G9a and GLP over-expression on H3K9me2. (**A**) The protein expression of G9a was detected after 6 h of G9a mRNA microinjection (G9a-mi). L, the G9a long isoform. S, the G9a short isoform. (**B**) The protein expression of GLP was detected after 6 h of GLP mRNA microinjection (GLP-mi). Rabbit Globin mRNA (Globin-mi) was microinjected as control. Actb (beta-actin) was used as a loading control. (**C**) The H3K9me2 state of zygotes at 12 h of IVF after mRNA microinjection. White arrow indicates the increased H3K9me2 of the male pronucleus, and red arrow indicates the low H3K9me2 level of the male pronucleus. Scale bar = 20 μm.

We next over-expressed G9a and GLP protein in mouse zygotes by mRNA microinjection. The protein level of G9a and GLP increased significantly at 6 h of microinjection as detected by western blotting (Fig. 4A,B). After G9a over-expression, the asymmetric H3K9me2 disappeared and both pronuclei showed an intensive immunostaining signal (indicated with white arrow, Fig. 4C). However, after GLP over-expression, the asymmetric H3K9me2 in zygotes still persisted (indicated with red arrow, Fig. 4C) although the immunostaining signal of the GLP over-expression group appeared to be more distinct than the control and rabbit Globin over-expression group.

### G9a over-expression did not increase H3K9me2 before 8 h of IVF

**Figure 5:**
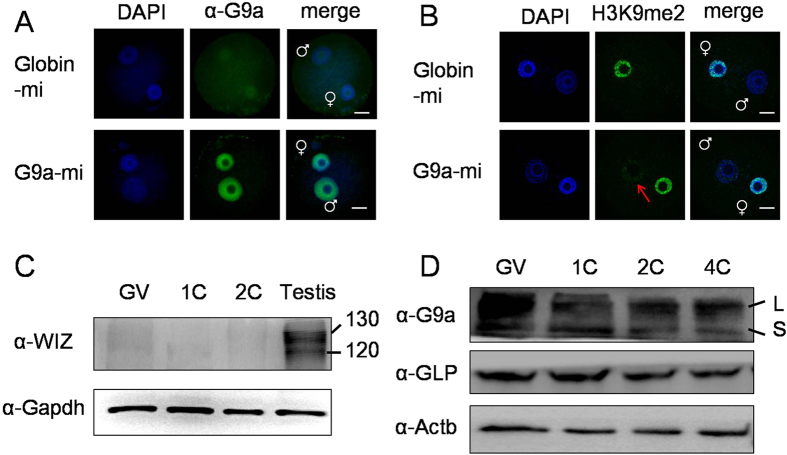
G9a and GLP may function independently without WIZ in mouse zygotes. (**A**) Immunostaining for the G9a in zygotes at 6 h of IVF after G9a mRNA microinjection. DNA was counterstained with DAPI. (**B**) The H3K9me2 state of zygotes at 8 h of IVF after G9a mRNA microinjection. Rabbit Globin mRNA (Globin-mi) was microinjected as control. White arrow indicates the increased H3K9me2 of the male pronucleus. Scale bar = 20 μm. (**C**) The expression of WIZ in GV oocytes, one-cell embryo (1C), two-cell embryo (2C) and testis. Gapdh was used as a loading control. (**D**). The expression of G9a and GLP in GV oocytes, one-cell embryo (1C), two-cell embryo (2C) and four-cell embryo (4C). Actb (beta-actin) was used as a loading control. L, the G9a long isoform. S, the G9a short isoform.

As mentioned above, a different mechanism for asymmetric H3K9me2 was apparent in zygotes before 8 h of IVF, as the H3K9me2 level in the male pronucleus showed no increase after cycloheximide treatment by this time point (Fig. 1A). We then investigated the change of H3K9me2 at the PN 3 stage after G9a mRNA microinjection. After over-expression, G9a protein already increased clearly at 6 h of IVF (Fig. 5A), but the H3K9me2 level of the male pronucleus still showed no increase at 8 h of IVF (Fig. 5B).

### WIZ showed no expression in mouse zygotes

In mammalian cells, the zinc finger protein WIZ targets G9a and GLP to the chromatin and mediates the G9a/GLP heteromeric complex-dependent H3K9 methylation as well as gene repression^34^,^35^. The Wiz/G9a/GLP tri-complex may protect G9a from degradation, and Wiz plays a major role in G9a/GLP heterodimer formation^20^. However, indications are that there may not be any expression of WIZ in zygotes and early embryos in the EST profile of NCBI (UniGene, Mm.274948). Our results also showed that there was almost no expression of WIZ protein in zygotes and early embryos, compared to the expression in testis (Fig. 5C). The expression of G9a and GLP proteins was stable during early embryo development (Fig. 5D).

## Discussion

**Figure 6:**
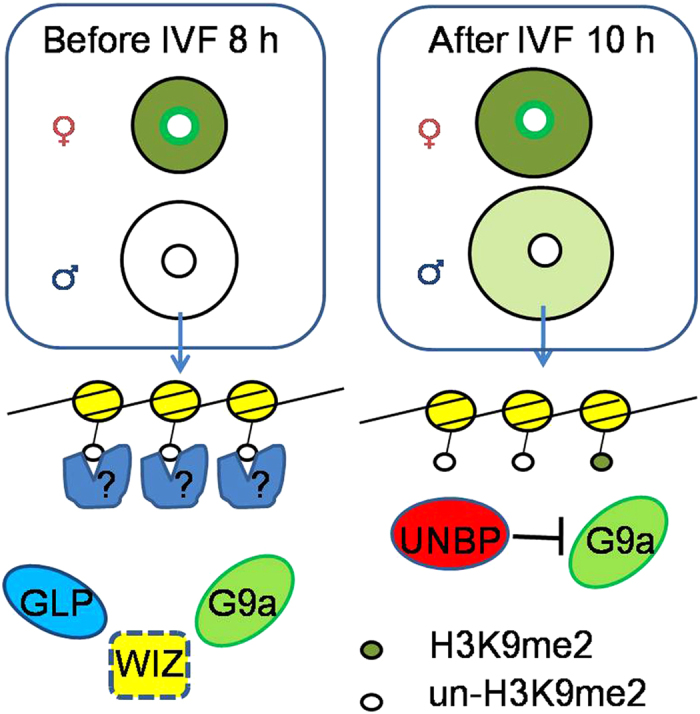
Schematic figure showing possible mechanism of asymmetric H3K9me2 in fertilized eggs. Certain unknown protein (?) prevents G9a from binding to H3k9 sites of the male pronucleus before 8 h of IVF. The unknown new born protein (UNBP) inhibits G9a catalysis after 10 h of IVF.

In this study, we investigated the alterations of H3K9me2 in mouse zygotes and the roles of G9a and GLP in these alterations. Our results suggest that there are two distinct mechanisms that regulate H3K9me2 in the male pronucleus of mouse zygotes (Fig. 6). Before 8 h of IVF, H3K9me2 of the male pronucleus remained at a low level, and the asymmetric H3K9me2 pattern of paternal and maternal genomes remained after cycloheximide treatment (Fig. 1A), which indicates that this asymmetric H3K9me2 pattern does not depend on new protein synthesis. After over-expression of G9a at 2 h of IVF, the asymmetric H3K9me2 pattern of paternal and maternal genomes also showed no change at 8 h of IVF (Fig. 5B). These results indicate that the asymmetric H3K9me2 was not caused by inhibition of the methyltransferase activity, but it was caused because the methyltransferase was prevented from binding to H3k9 sites by certain unknown protein(s).

After 10 h of IVF, the mechanism of the asymmetric H3K9me2 pattern between paternal and maternal genomes depends on new protein synthesis, as summarized in a previous study^24^. The two mechanisms of transfer between 8–10 h of IVF, and the synthesis of unknown new protein(s) cannot totally inhibit the methyltransferase activity in time. This results in the low level of *de novo* H3K9me2 appearance in the male pronucleus. In this study, we confirmed the function of new protein synthesis inhibition on H3K9me2 of the male pronucleus by cycloheximide and puromycin treatment (Fig. 1B). Cycloheximide^36^ and puromycin^37^ are two different protein synthesis inhibitors, and both can cause the disappearance of asymmetric H3K9me2 patterns in mouse zygotes.

Between 8–10 h of IVF the mouse zygotes are in the S phase and DNA replication is in progress. We thus investigated whether DNA replication and cell cycle regulation were involved in the regulation of H3K9me2 in mouse zygotes. Aphidicolin and roscovitin inhibit DNA replication and cyclin-dependent kinase (CDK), respectively. The results of aphidicolin and roscovitin treatment indicate that H3K9me2 of the male pronucleus did not significantly change when either DNA replication or cyclin/cyclin-depended kinase is inhibited (Fig. 1B). This indicates that DNA replication and cell cycle regulation may not be involved in the regulation of H3K9me2 in mouse zygotes.

G9a and GLP form a heteromeric complex to mediate H3K9 methylation as well as gene repression in somatic cells^34^,^35^. However, in mouse zygotes G9a and GLP may function independently without WIZ expression (Fig. 5C). Our results indicate that G9a, but not GLP, is involved in the regulation of asymmetric H3K9me2 in mouse zygotes. And G9a is the methyltransferase which induces the appearance of H3K9me2 in the male pronucleus of the zygote treated with cycloheximide. Knock-down by G9a antibody or siRNA microinjection and over-expression of G9a by mRNA microinjection both can influence the H3K9me2 of the male pronucleus (Figs 2B,3 and 4). The results indicate that G9a is involved in the regulation of asymmetric H3K9me2 in mouse zygotes.

Post-translational modification of histone proteins in chromatin and DNA methylation are two major mechanisms involved in epigenetic modifications of genomes, which are regulated by distinct, but coupled, pathways^38^. G9a/GLP complexes independently mediate H3K9 and DNA methylation to silence transcription^21^. PGC7 binds histone H3K9me2 to block the activity of the Tet3 methylcytosine oxidase in the maternal genome as well as at certain imprinted loci in the paternal genome^39^, thereby protecting against conversion of 5 mC to 5 hmC in early embryos^40^. The parental pronuclei have asymmetric reprogramming capacities and the reprogramming factors reside predominantly in the male pronucleus^41^. Our results indicate that the alterations of H3K9me2 may play a role in the chromatin remodeling and cell reprogramming.

In the current study, we used mouse zygotes from IVF instead of natural fertilized zygotes for experimental feasibility, as we can determine with greater accuracy the fertilization time by using an IVF system. A previous study showed that there was no difference in the H3K9 methylation pattern in *in vivo* and IVF mouse zygotes^42^. Taken together, the study of epigenetic regulation in IVF zygotes may benefit improvement of embryo culture and treatment of female infertility. Understanding the epigenetic regulation mechanisms in mouse zygotes will also benefit research of chromatin remodeling and cell reprogramming, and it is important for understanding the mechanism of gene expression regulation and cell differentiation in embryo development.

## Materials and Methods

### Oocyte collection and culture

All experiments and methods were carried out in accordance with the protocols approved by the Animal Research Committee of the Institute of Zoology, Chinese Academy of Sciences, China. Mice were housed in 12-hour alternating light/dark cycles, with free access to water and food. The GV stage oocytes were isolated from ovaries of 6- to 8-week-old female ICR mice and cultured in M16 medium (Sigma) under paraffin oil at 37 °C, 5% CO_2_ in air for up to 12 h.

### IVF and embryo culture

Spermatozoa were collected from the caudal epididymis of adult ICR males, and pre-incubated in human tubal fluid (HTF) medium for 1 h in an atmosphere of 5% CO_2_, 95% air at 37 °C. Superovulated metaphase II-arrested (M II) oocytes were collected from the ampullae of the oviducts in HTF medium, 14–15 h of hCG administration. The oocytes were inseminated with capacitated spermatozoa. Two hours after insemination, fertilized eggs were washed and cultured in KSOM medium^43^, and collected at the times indicated. All media were pre-incubated for at least 2 h in an atmosphere of 5% CO_2_, 95% air at 37 °C. The zygotes were collected at 4, 6, 8, 10 and 12 h of IVF, respectively.

### Reagent treatments

Fertilized eggs were transferred into KSOM medium containing cycloheximide (CHX, 15 μg/mL. 01810), puromycin (Puro, 1 mg/mL, P8833), BIX 01294 (BIX, 5 μM, B9311), roscovitin (ROS, 200 μM, R7772) or aphidicolin (Aphi, 3 μg/mL, A0781), and collected at the times indicated. Fertilized eggs cultured in normal KSOM medium were used as control group. All reagents were purchased from Sigma-Aldrich Co. and stored following the product instructions.

### Plasmid construction and mRNA synthesis

The full-length G9a (GenBank: NM_145830) and that of GLP (GenBank: NM_001012518.3) CDS were cloned to pcDNA3.1 vector. The plasmids were linearized by NotI and purified by gel extraction kit (Promega). T7 highyield capped RNA tanscription kit and Poly (A) Tailing Kit (Ambion) were used for producing capped and tailed mRNA, and then the mRNA was purified with RNeasy cleanup kit (Qiagen). The concentration of G9a and GLP mRNA was determined with a Beckman DU 530 Analyzer and then diluted to a high concentration (1.5 mg/mL) for over-expression.

### Microinjection of siRNA, antibodies or mRNA

Microinjection was performed using an Eppendorf microinjector and completed within 1 h. The siRNAs were diluted to 20 μM and microinjected into the cytoplasm of GV stage oocytes. After microinjection, the GV stage oocytes were cultured for 12 h in M16 medium supplemented with 0.2 mM IBMX to maintain oocytes at the GV stage, and then transferred into normal M16 medium for 12 h for *in vitro* maturation. The matured M II oocytes were selected and intracytoplasmic sperm injection (ICSI) was performed; culture followed in KSOM medium containing cycloheximide. The G9a siRNA sequence is 5′-AUACGAAUCACAUCGAUGUGCUUGU-3′, GLP siRNA sequence is 5′-AAAUGCAGCCGCUUGCUCAGCUCCA-3′, and scrambled control siRNA sequence is 5′-UUUCUCCAUACCAUUUCAUCC AUCC-3′ (synthesized by Life Technology). G9a (sc-22877, Santa Cruz) and GLP (sc-68165, Santa Cruz) antibodies or mRNA solution were microinjected into cytoplasm of fertilized eggs at 2 h of IVF. The rabbit Globin mRNA was used as negative control.

### Quantitative real time-PCR

Approximately 60 oocytes for each group were used to extract RNA for reverse transcription reactions. Expression level of G9a and GLP was validated by quantitative real-time PCR analysis (Roche 480) according to the manufacturer’s instructions. Primers for G9a (PrimerBank ID 22164772a1), GLP (PrimerBank ID 26352233a1) and beta-actin (actb, PrimerBank ID 6671509a1) were synthesized by Sangon Biotech (Shanghai) Co., Ltd. The experiments were repeated at least 3 times.

### Western blot analysis

About 200 mouse oocytes or zygotes or 30 μg testis protein per sample were mixed with SDS sample buffer and boiled for 5 min at 100 °C for SDS-PAGE. Western blotting was performed as described previously^44^ using the antibody dilution anti-G9a (3306S, Cell Signaling Technology) at 1:1000; anti-GLP (ab41969, Abcam) at 1: 1000; anti-Actb (BS6007, Bioward) at 1: 1000; anti-Gapdh (MB001, Bioward) at 1: 1000. The membranes were subsequently incubated with HRP-conjugated secondary antibodies (1:2000; ZB2301 and ZB2305, Zhongshan Golden Bridge Biotechnology) for 1 h at 37 °C. Protein bands were detected using Thermo Supersignal West Pico chemiluminescent substrate.

### Immunofluorescence and confocal microscopy

Zygotes were fixed for 1 h in 3.7% paraformaldehyde in PBS, and permeabilized with 0.5% Triton X-100 in PBS for 20 min at room temperature. Then the zygotes were incubated at 4 °C overnight with primary antibodies (H3K9me2 at 1:200, 07–212, Millipore; G9a at 1:200, 3306S, Cell Signaling Technology), and then incubated for 1 h with a secondary AlexaFluor 488-conjugated antibody or AlexaFluor 594-conjugated antibody (1:1000, A11008 and A11012, Life Technology). DNA was stained for 20 min with DAPI (4, 6-diamidino- 2-phenylindole). Fluorescence was detected using a Zeiss LSM780 laser-scanning confocal microscope.

### Data analysis

All experiments were repeated at least three times. Statistical analysis was performed using SPSS. Data were expressed as mean ± S.E.M. and P < 0.01 was considered as statistically significant.

## Additional Information

**How to cite this article**: Ma, X. *et al.* The Dynamics and Regulatory Mechanism of Pronuclear H3k9me2 Asymmetry in Mouse Zygotes. *Sci. Rep.* **5**, 17924; doi: 10.1038/srep17924 (2015).
